# The risk of postoperative deterioration of non-cardiac surgery patients with ICU referral status who are admitted to the regular ward: a retrospective observational cohort study

**DOI:** 10.1186/s13037-021-00283-9

**Published:** 2021-02-21

**Authors:** David Moore, Matthew L. Durie, Sohail Bampoe, Luke Buizen, Jai N. Darvall

**Affiliations:** 1 Department of Anaesthesia and Pain Management, Royal Melbourne Hospital, Grattan Street, Melbourne, VIC 3050 Australia; 2 Centre for Perioperative Medicine, UCL Division of Surgery and Interventional Science, University College London, London, UK; 3 Melbourne EpiCentre, University of Melbourne, Melbourne, Australia; 4 Centre for Integrated Critical Care, University of Melbourne, Melbourne, Australia

**Keywords:** Anaesthesia safety, Intensive care, Length of stay

## Abstract

**Background:**

Higher-risk surgical patients may not be admitted to the intensive care unit due to stable immediate post-operative status on review. The outcomes of this cohort are not well described. Our aim was to examine the subsequent inpatient course of intensive care unit -referred but not admitted surgical patients.

**Methods:**

All patients aged ≥18 years who were referred but not admitted for post-operative management in a tertiary metropolitan intensive care unit following non-cardiac surgery between 1/7/2017 and 30/6/2018 were eligible for inclusion in this retrospective observational cohort study. Primary outcome was Medical Emergency Team activation. Secondary outcomes included unplanned intensive care unit admission; length of stay; and 30-day mortality. Risk of serious complications and predicted length of stay were calculated using the National Surgical Quality Improvement Program scoring tool.

**Results:**

Fifteen of 60 patients (25%) had a MET-call following surgery, eight (13%) patients required unplanned intensive care unit admission, with median (IQR) time to Medical Emergency Team call 9 (6–13) hours. No patients died within 30-days. There was no significant difference between mean National Surgical Quality Improvement Program predicted and actual length of stay; after adjustment, National Surgical Quality Improvement Program predicted risk of serious complications was associated with unplanned intensive care unit admission (OR [95% CI] = 1.08 [1.00–1.16], *p* = 0.04), although not Medical Emergency Team calls.

**Conclusions:**

Post-operative deterioration occurs frequently, and early, in a cohort of high-risk surgical patients initially assessed as being safe for ward care. Changes to current triage models for post-operative intensive care unit admission may reduce the impact of complications in this high-risk group.

**Supplementary Information:**

The online version contains supplementary material available at 10.1186/s13037-021-00283-9.

## Background

Post-operative ward deterioration defines a patient cohort admitted to the intensive care unit (ICU) at risk of poor outcomes, with the majority of peri-operative complications disproportionately arising in a small number of high-risk patients [1, 2]. Prior studies have demonstrated considerable excess mortality when planned post-operative ICU admission does not proceed, especially among those patients who are ultimately admitted to ICU following a deterioration after initial ward based care [3–5]. As few as 15% of these high-risk patients are electively admitted to the ICU post-operatively, despite accounting for up to 80% of post-operative deaths [6]. Identification of this higher-risk patient group may thus allow earlier intervention, to potentially improve both patient outcomes and ICU resource allocation [2].

There remains considerable uncertainty, however, in how to best risk-stratify patients who will benefit most from routine post-operative ICU admission [1]. The most widespread surgical risk-scoring system, the American College of Surgeons National Surgical Quality Improvement Program (NSQIP) Surgical Risk Calculator, is based on a database containing the outcomes of several million surgeries, and provides mortality and major morbidity estimates with ongoing calibration against actual patient outcomes [7]. Unfortunately, such formal risk scoring is not commonly incorporated in triage decision making for post-operative ICU admission. Instead, the commonly performed “bedside” ICU review of patients in the immediate post-operative period may be confounded by optimised patient physiology by attending anaesthetists. High-risk patients may subsequently suffer deterioration in the ward, however the outcomes of patients who are initially referred for post-operative ICU, but not admitted due to perceived safety for ward discharge, are not known.

The aim of this study, therefore, was to examine the subsequent course of a cohort of post-operative patients referred but not admitted to ICU. We hypothesized that Medical Emergency Team (MET) activation and unplanned ICU admission would be common in this population. We secondarily hypothesized that clinical deterioration would occur early in the post-operative course, and that NSQIP scoring could be used to predict patients at risk of deterioration.

## Methods

We performed a retrospective, observational cohort study at the Royal Melbourne Hospital, a tertiary referral metropolitan health service with a surgical caseload of > 12,000 patients annually. Patients aged ≥18 years undergoing non-cardiac surgery between 1/7/2017 and 30/6/2018 who were initially reviewed for post-operative ICU care but discharged to the ward were eligible for inclusion. Patients were identified from the hospital internal “REFER-ICU” database, inclusion in this database is obligatory prior to consideration for ICU admission. Post-operative patients referred for ICU review were attended in the post-anaesthesia care unit by a senior ICU registrar, and subsequently discussed with the admitting intensivist; no formal risk stratification tools were used in triage decisions.

Perioperative data collected from the medical record included patient age, sex, operation type and urgency (elective vs. emergency), American Society of Anesthesiology (ASA) score, length of hospital post-operative stay, death in hospital, as well as variables required to generate NSQIP predicted length of stay (LOS), risk of death and risk of serious complications: cardiac arrest, myocardial infarction, pneumonia, progressive renal insufficiency, acute renal failure, pulmonary embolism, deep venous thrombosis, unplanned return to the operating theatre, deep incisional/organ space surgical site infection, systemic sepsis, unplanned intubation, urinary tract infection, and wound disruption (definitions in Supplementary Table 1). The NSQIP model uses a single round of multivariable imputation for missing data points, in the event of missing NSQIP data, perioperative risk and length of stay predictions were generated using remaining variables [8].

The primary outcome was MET call activation after post-operative ward admission. Secondary outcomes were unplanned ICU admission, in-hospital mortality, and hospital LOS. We further compared the actual LOS with that predicted by NSQIP scoring.

### Statistical analysis

Demographic and outcome data were summarised as number (%), mean (standard deviation [SD]) or median (interquartile range [IQR]) in the case of non-parametric data. NSQIP predicted and actual LOS were compared using difference in medians. Multivariable logistic regression was used to predict MET activation and unplanned ICU admission. Gender, NSQIP predicted LOS and NSQIP predicted risk of serious complications were included in the initial model. All variables with a *p*-value < 0.20 in univariable analysis were considered for inclusion into the model. As NSQIP predictors were highly correlated (pearson correlation = 0.89, *p*-value < 0.001), only the most statistically significant predictor of MET call (NSQIP predicted LOS) was included in the model. Backwards elimination was used to remove parameters with a type 3 Wald *p*-value > 0.05, thus NSQIP predicted LOS was eliminated from the model, with only gender remaining (*p*-value = 0.017).

## Results

Sixty post-operative patients fulfilling inclusion criteria were identified from 3068 total ICU (surgical and non-surgical) referrals during the 12-month study period. Median (IQR) age was 67 (50–77) years, 33 (55%) of patients presented after emergency surgery, 54 (90%) of patients had an ASA score ≥ 3 (Table 1).

**Table 1:** Baseline demographics of study patients

Variable	Total *N* = 60
Age (years), median (IQR)	67 (50–77)
BMI (kg/m^2^), median (IQR)	26 (24–31)
Male, n (%)	31 (52%)
Emergency surgery, n (%)	33 (55%)
Surgical specialty, n (%)	
General surgery	21 (35%):
Upper GI	9 (15%)
Colorectal	6 (10%)
Other specialist general surgery	6 (10%)
Orthopaedics	10 (17%)
Urology	8 (13%)
Neurosurgery	7 (12%)
Vascular	3 (5%)
Head & Neck	3 (5%)
Renal Transplant	2 (3%)
Plastics	2 (3%)
Upper GI Endoscopy	2 (3%)
Trauma	1 (2%)
Cardiology	1 (2%)
ASA score	
1	0 (0%)
2	6 (10%)
3	48 (80%)
4	5 (8%)
5	1 (2%)
NSQIP predicted % serious complication, mean (SD)	19.9% (11.2%)
NSQIP predicted % mortality, median (IQR)	2.8% (0.8–11.0%)

*BMI* Body mass index; *ASA* American Society of Anesthesiologists score; *NSQIP* National Surgical Quality Improvement Program.

**Fig. 1:**
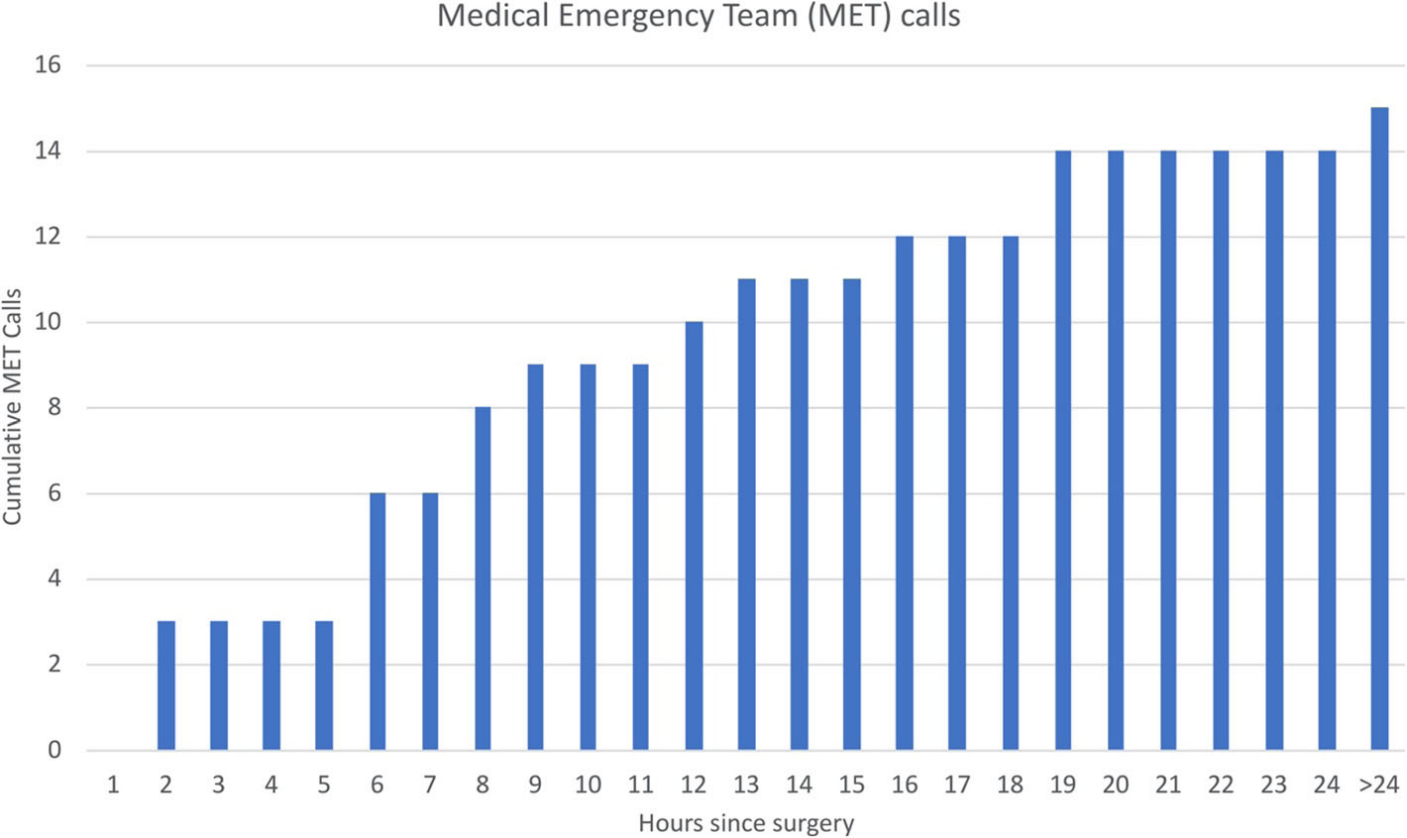
Timing of Medical Emergency Team (MET) calls

Fifteen (25%) of patients deteriorated and required MET call activation; median (IQR) time to first MET call was 8.8 (6.1–13.3) hours, with 14 of 15 MET calls activated in the first 24 h (Fig. 1).

MET calls were activated for post-operative hypotension in eight patients, tachycardia/chest pain in four patients, and respiratory distress/desaturation in a further three patients. Eight (13%) patients were subsequently admitted to ICU, six of these admissions were following a MET call (Table 2).

**Table 2:** Post-operative outcomes

Variable	Total *N* = 60	Male *N* = 31	Female *N* = 29
MET call activation	15 (25%)	4 (12.9%)	11 (37.9%)
Unplanned ICU admission	8 (13.3%)	1 (3.2%)	7 (24.1%)
NSQIP predicted LOS, days, median (IQR)	7.5 (3.3–10.3)	7.5 (3.3–9.3)	7.5 (3.5–12.0)
Actual LOS, days, median (IQR)	7.5 (3.5–13.0)	7.0 (4.0–12.5)	8.0 (4.0–13.0)
30-day mortality, n (%)	0 (0%)	0 (0%)	0 (0%)

*NSQIP* National Surgical Quality Improvement Program; *PACU* Post-anaesthesia care unit; *MET* Medical emergency team.

There were no deaths within 30 days of surgery, median (IQR) length of stay was 7.5 (3.5–13.0) days.

NSQIP predicted and actual post-operative LOS were not different for the total cohort (mean [SD] difference 2.8 [11.4] days, *p* = 0.07), nor did predicted LOS differ between patients who had a MET call and those who did not (mean [SD] 10.0 [4.7] vs. 7.2 [5.7] days, *p* = 0.09), or between patients admitted and not admitted to ICU (mean [SD] 10.4 [4.2] vs. 7.6 [5.6] days, *p* = 0.06) (Table 3).

**Table 3:** Association between outcomes and NSQIP predicted and actual length of stay

	MET Call (***N*** = 15)		Unplanned ICU Admission (***N*** = 8)	
	Yes (***N*** = 15)	No (***N*** = 45)	Yes (***N*** = 8)	No (***N*** = 52)
Predicted LOS, days	10.0 (4.7)	7.2 (5.7)	9.8 (7.8–12.0)	7.3 (3.0–10.0)
Mean difference (95% CI)	2.8 (− 5.9–0.4)			
*P*-Value	0.09		0.06	
	**Yes (*N*** **= 15)**	**No (*N*** **= 45)**	**Yes (*N*** **= 8)**	**No (*N*** **= 52)**
Actual LOS, days	13.9 (11.3)	10.2 (13.6)	12.5 (9.5–14.5)	7.0 (3.0–12.0)
Mean difference (95% CI)	−3.7 (−11.3–4.0)			
*P*-Value	0.34		0.04	

*LOS* Length of stay, either mean (SD) or median (IQR). *MET* Medical Emergency Team; *ICU* Intensive Care Unit.

There was also no association between NSQIP predicted risk of serious complications and MET calls (*p* = 0.10), although risk of serious complications was associated with unplanned ICU admission (OR [95% CI = 1.08 [1.00–1.16], *p* = 0.04). (Table 4). Actual LOS was longer in patients admitted to ICU (median [IQR] 7.0 [3.0–12.0] vs. 12.5 [9.5–14.5] days, *p* = 0.04).

**Table 4:** Univariable analyses: MET call, unplanned ICU admission Odds Ratio (95% CI)

	Odds Ratio (95% CI)	***P***-value
MET Call		
Gender (Female vs Male)	4.8 (1.3 – 7.2)	0.02
NSQIP Predicted length of stay	1.09 (0.99 – 1.21)	0.09
NSQIP Predicted risk of serious complications	1.05 (1.00 – 1.10)	0.10
Unplanned ICU admission		
Gender (Female vs Male)	9.5 (1.1 – 83.3)	0.04
NSQIP Predicted length of stay	1.09 (0.96 – 1.24)	0.18
NSQIP Predicted risk of serious complications	1.08 (1.00 – 1.16)	0.04

## Discussion

### Key findings

In this observational cohort study, we found that one-quarter of patients initially assessed after surgery as safe for ward care had a clinically significant deterioration leading to a MET call, which occurred early in the post-operative course (median 9 h). Eight of 60 patients were also subsequently admitted emergently to the ICU. There were no significant differences between predicted and actual LOS in either the total cohort or the subset of patients with a MET call/unplanned ICU admission, suggesting validity of NSQIP scoring in this patient cohort. NSQIP predicted risk of serious complications was associated with unplanned ICU admission. However, this did not differ between patients with and without a MET call. Both sexes had similar baseline risk profiles, however both MET call and unplanned ICU admission were more common in female patients, with longer lengths of stay; this result is hypothesis generating and requires investigation in a larger data set.

### Relationship to prior literature

There are no large randomised controlled trials comparing routine post-operative ICU to ward based care for high-risk surgical patients. Observational studies, however, demonstrate increased risk of deterioration in high-risk patients who do not initially receive a higher intensity of post-operative management [2, 9].. A Scottish study incorporating > 500,000 surgical patients demonstrated higher mortality and a greater requirement for organ supports in patients with delayed admission to ICU after initial ward care, compared to those directly admitted [4]. Other studies, however, have questioned the role of routine post-operative ICU admission. The STARsurg collaborative did not demonstrate any improvement in 30-day mortality following routine ICU admission for patients undergoing major gastrointestinal or liver surgery [10]. Similarly, the International Surgical Outcomes Study (ISOS) group showed no association between mortality and ICU admission after elective surgery, even for high-risk patients [11]. Disparate findings between these studies are likely influenced by differences in baseline patient characteristics, risk profiles, and variations in admission criteria and post-operative care provided in individual ICUs. Our study design is unique in examining outcomes in a patient group that was deemed high-risk enough for ICU referral yet initially underwent ward-based management, rather than assessing the role of routine post-operative ICU admission. As such, our study offers novel insights into the outcomes of these higher-risk patients, if not initially admitted to ICU.

A 2010 European study (not surgical patient specific) examining reasons for refusal of ICU admission investigated > 8000 ICU triage decisions in 11 hospitals across seven countries. This demonstrated a 90-day mortality of 18% for patients assessed as “too well” for ICU as admission [12]. Similarly, the baseline characteristics of our cohort confirm their status as a high-risk group, with mean NSQIP risk of serious complications of 20%. ICU bed availability may be another factor influencing triage decisions. A 2012 European study found an increased mortality in patients who had initially been declined ICU admission due to capacity constraints, but subsequently admitted after re-referral [13]. We were unable to examine ICU bed capacity at the time of referral, thus we cannot exclude the possibility that this influenced decisions regarding post-operative ward admission.

An important finding of this study was that significant clinical deterioration occurred very early in the post-operative period (median 9 h) after patients had been assessed as safe for ward care. Thus, rather than representing later post-operative complications, such as wound infection or pneumonia, this temporal association implies that early deterioration was related to the initial surgery, questioning the model of ICU triage occurring in the PACU. Furthermore, this may suggest that benefit can be derived for higher-risk patients with a greater level of monitoring in a higher-dependency area immediately following surgery, and that such benefit does not require many days of admission prior to “pay-off”.

In our cohort, NSQIP risk scoring did not differentiate between which patients went on to have a MET call activation, however on adjusted analysis this was associated with unplanned ICU admission. Future research should seek to examine this association in an unselected group of surgical patients; it is possible that a threshold may be identified at which routine ICU admission is deemed cost- and resource-effective. It also cannot be inferred from this observational study if such elective ICU admission would have prevented either ward deterioration or the increased LOS observed in those patients with subsequent unplanned ICU admission. Future research should examine the outcomes of routinely ICU-admitted post-operative patients based on objective risk scoring criteria and aim to identify which specific interventions or components of higher level post-operative care are associated with improved outcomes.

An unexpected finding from this study was the identification of sex differences for MET call/ICU admission and LOS, with worse outcomes observed in female patients, despite similar NSQIP risk profiles. This observation is unusual compared with prior studies, and likely represents a chance finding [14]. Larger studies could confirm whether this difference is real.

Strengths of this study include the inclusion of all referred post-operative patients over a 12-month period in a major metropolitan hospital, across a wide range of surgical specialities, thus enhancing external validity. The measured outcomes of MET activation, unplanned ICU admission and mortality are clinically significant and generalisable between health services. Limitations of our study include the single centre design, with some components of peri-operative care perhaps differing between institutions, limiting applicability. The retrospective, observational design also means causative effects cannot be explored.

## Conclusion

A high proportion of patients who are assessed as safe for ward care, following referral for post-operative ICU admission, suffer significant clinical deterioration early in the post-operative period. These patients have a high rate of ultimate ICU admission, leading to increased length of stay. In this cohort, the NSQIP risk scoring system was associated with patients who subsequently had an unplanned ICU admission, but not a MET call. Future research should seek to identify threshold risk scores to guide routine ICU admission in high-risk surgical patients.

## Supplementary Information

**Additional file 1: Table S1**. Definition of NSQIP variables.

## Data Availability

The datasets used and/or analysed during the current study are available from the corresponding author on reasonable request.
