# Supplementary material for: The risk of postoperative deterioration of non-cardiac surgery patients with ICU referral status who are admitted to the regular ward: a retrospective observational cohort study
Source: Patient Saf Surg. 2021 Feb 21;15:10. doi: 10.1186/s13037-021-00283-9 (PMC7897383; doi:10.1186/s13037-021-00283-9)
Supplement: Supplementary file 1 — Additional file 1: Table S1. Definition of NSQIP variables. [file 13037_2021_283_MOESM1_ESM.docx]

**Supplementary Table 1: Definition of NSQIP variables**

© 2007-2020, American College of Surgeons National Surgical Quality Improvement Program. All rights Reserved

| **Variable** | **Definition** |
| --- | --- |
| Cardiac arrest | The absence of cardiac rhythm or presence of a chaotic cardiac rhythm requiring the initiation of CPR, which includes chest compressions. |
| Myocardial infarction | ECG changes, new elevation in troponin, or physician diagnosis. |
| Pneumonia | Infection of the lungs, diagnosed using both radiologic (i.e., infiltrate, consolidation or opacity, cavitation) and clinical (e.g., fever, leukopenia / leukocytosis, culture results, patient symptoms) criteria. |
| Progressive renal insufficiency | A rise in creatinine of >2 mg/dl from preoperative value, but with no requirement for dialysis. |
| Acute renal failure requiring dialysis | A patient who did not require dialysis preoperatively, worsening of renal dysfunction postoperatively requiring hemodialysis, peritoneal dialysis, hemofiltration, hemodiafiltration, or ultrafiltration. |
| Venous thromboembolism / blood clot | The identification of a new thrombus within the venous system, described in studies as present in the superficial or deep venous systems but requires therapy. This diagnosis is confirmed by a duplex, venogram, CT scan or other imaging modality, AND the patient requires treatment with anticoagulation therapy and/or placement of a vena cava filter or clipping of the vena cava. |
| Return to operating room | Return to the operating room for additional surgery that was not planned at the time of the initial surgery. |
| Surgical site infection | Superficial Incisional SSI: infection that involves only skin or subcutaneous tissue of the incision. It also includes either: purulent drainage, positive culture, signs/symptoms of infection and the incision is deliberately opened by the surgeon or diagnosis by the attending physician.  Deep Incisional SSI: infection that appears to be related to the operation and involves deep soft tissues (for example, fascial and muscle layers) of the incision. It also includes either: purulent drainage, spontaneous dehiscence, deliberate opening by the surgeon, abscess involving the deep incision, or diagnosis by the attending physician.  Organ Space SSI: infection that involves any part of the anatomy (for example, organs or spaces), other than the incision, which was opened or manipulated during an operation. It also includes either: purulent drainage, positive culture, abscess, or diagnosis by the attending physician. |
| Urinary tract infection | Bladder infection, diagnosed using a combination of clinical symptoms and laboratory confirmation (e.g., urine culture, pyuria, positive dipstick) or initiation of appropriate antimicrobial therapy. |
| Systemic sepsis | Any of the following occurring within 48 hours prior to surgery: Systemic Inflammatory Response Syndrome (SIRS), Sepsis, Septic Shock |
| Functional Status | The best functional status/level of self-care demonstrated by the patient within the 30 days prior surgery.  Independent: The patient does not require assistance from another person for any activities of daily living. This includes a person who is able to function independently with prosthetics, equipment, or devices  Partially dependent: The patient requires some assistance from another person for activities of daily living.  Totally dependent: The patient requires total assistance for all activities of daily living. |
| American Society of Anesthesiology Class | ASA 1: Normal healthy patient.  ASA 2: Patient with mild systemic disease.  ASA 3: Patient with severe systemic disease.  ASA 4: Patient with severe systemic disease that is a constant threat to life.  ASA 5: Moribund patient who is not expected to survive without the operation. |
| Steroid use for chronic condition | Regular administration of oral or parenteral corticosteroid medications or immunosuppressants for a chronic medical condition, within the 30 days prior to surgery, or at the time the patient is being considered as a candidate for surgery. A one-time pulse, limited short course, or a taper of less than 10 days duration would not qualify. Long-interval injections of long-acting agents would qualify. |
| Ascites within 30 days of surgery | The presence of fluid accumulation in the peritoneal cavity noted on physical examination, abdominal ultrasound, or abdominal CT/MRI within 30 days prior to surgery. Documentation must state either active or a history of liver disease or must state secondary to malignancy. |
| Systemic sepsis within 48 hours prior to surgery | Any of the following occurring within 48 hours prior to surgery: Systemic Inflammatory Response Syndrome (SIRS), Sepsis, Septic Shock |
| Ventilator dependent | A patient requiring ventilator-assisted respiration at any time during the 48 hours preceding surgery. This does not include the treatment of sleep apnea with CPAP. |
| Disseminated cancer | The patient has a primary cancer that has metastasized to a major organ AND meets at least one of the following:  active treatment for the cancer within one year of the surgery date. If the surgical procedure is the treatment for the metastatic cancer, answer "Yes”  The patient has elected not to receive treatment for the metastatic disease  the patient's metastatic cancer has been deemed untreatable  Report the following cancers as Disseminated Cancer: Acute Lymphocytic Leukemia (ALL), Acute Myelogenous Leukemia (AML), and Stage IV Lymphoma.  Do not report the following as Disseminated Cancer: Chronic Lymphocytic Leukemia (CLL), Chronic Myelogenous Leukemia (CML), Stages I through III Lymphomas or Multiple Myeloma. |
| Diabetes | The individual requires daily dosages of exogenous parenteral insulin or an oral hypoglycemic agent to prevent a hyperglycemia. A patient is not included if diabetes is controlled by diet alone. |
| Hypertension requiring medication | The patient has a diagnosis of HTN in the medical record and will require antihypertensive medication(s) within 30 days prior to surgery. |
| Congestive Heart Failure in 30 days prior to surgery | Only newly diagnosed CHF within the previous 30 days or a diagnosis of chronic CHF with signs or symptoms of CHF in the 30 days prior to surgery fulfills this definition |
| Dyspnea | The patient's dyspnea status when they were in their usual state of health, prior to the onset of the acute illness, within the 30 days prior to the time the patient is being considered a candidate for surgery. |
| Current smoker within 1 year | The patient has smoked cigarettes in the year prior to admission for surgery. Patients who smoke cigars or pipes or use chewing tobacco are not included. |
| History of Severe COPD | Chronic obstructive pulmonary disease (such as emphysema and/or chronic bronchitis) resulting in one or more of the following:  Functional disability from COPD (for example, dyspnea, inability to perform ADLs)  Hospitalization in the past for treatment of COPD  Chronic bronchodilator therapy with oral or inhaled agents  FEV1 of <75% of predicted  Do not include patients whose only pulmonary disease is asthma  Do not include patients with diffuse interstitial fibrosis or sarcoidosis |
| Dialysis | Acute or chronic renal failure requiring treatment with peritoneal dialysis, hemodialysis, hemofiltration, hemodiafiltration, or ultrafiltration within 2 weeks prior to surgery. If a patient requires dialysis, but refuses it, the answer to this variable will be "Yes." |
| Acute renal failure | A clinical condition associate with rapid decline of kidney function. The patient meets one of the following:  Increased BUN on two measurements AND two Cr results > 3mg/dl  Surgeon or physician has documented Acute Renal Failure AND one of the following:   - - Increased BUN on two measurements   - Two Cr results > 3mg/dl |
